# Do Tobacco Companies Have an Incentive to Promote “Harm Reduction” Products?: The Role of Competition

**DOI:** 10.1093/ntr/ntad014

**Published:** 2023-01-24

**Authors:** David T Levy, Frances Thirlway, David Sweanor, Alex Liber, Luz Maria Sanchez-Romero, Rafael Meza, Clifford E Douglas, K Michael Cummings

**Affiliations:** Oncology Department, Lombardi Comprehensive Center, Georgetown University, Washington, DC, USA; Sociology Department, University of York, Heslington, York, UK; Centre for Health Law, Policy and Ethics, University of Ottawa, Ottawa, Ontario, Canada; Oncology Department, Lombardi Comprehensive Center, Georgetown University, Washington, DC, USA; Oncology Department, Lombardi Comprehensive Center, Georgetown University, Washington, DC, USA; Department of Epidemiology, University of Michigan School of Public Health, Ann Arbor, MI, USA; Department of Health Management and Policy, University of Michigan School of Public Health, Ann Arbor, MI, USA; Department of Psychiatry and Behavioral Sciences, Medical University of South Carolina, Charlestown, SC﻿, USA

## Abstract

**Introduction:**

Some cigarette companies have started to talk about replacing cigarettes with less harmful alternatives, which might include nicotine vaping products (NVPs), heated tobacco products (HTPs), and oral nicotine delivery products. We consider market competition as a primary driver of whether cigarette companies follow through on their stated intentions.

**Aims and Methods:**

We focus on the behavior of cigarette companies in the United States. We compare competition in the pre- and post-2012 time periods, analyze the impact of the growth in NVPs on smoking prevalence and cigarette company profits, and examine the potential future role of competition.

**Results:**

Since 2006, consumers have broadened their use of non-combustible nicotine delivery products (NCNDPs) to include, inter alia, NVPs, HTPs, and oral nicotine pouches. U.S. cigarette companies have acquired major stakes in each of these product categories which corresponds to a period of rapidly declining adult smoking prevalence, especially among younger adults (ages 18–24 years). The shifting dynamics of the nicotine product marketplace are also reflected in cigarette company stock prices. While cigarette companies are likely to promote HTPs and nicotine delivery products over NVPs, their incentives will be directly related to competition from independent firms, which in turn will depend on government regulation.

**Conclusions:**

Although cigarette companies will back alternatives to combusted tobacco when threatened by competition, the prospects for their lasting conversion to NCNDPs will depend on the extent of such competition, which will be influenced by government regulation of tobacco products.

**Implications:**

Regulations that limit competition from independent firms while also protecting cigarette company profits risk slowing or even reversing recent declines in smoking, especially among youth and young adults. Regulations that reduce the appeal and addictiveness of combusted tobacco products, such as higher cigarette taxes or a reduced nicotine standard, will encourage smokers to quit and/or switch to less harmful non-combusted forms of tobacco. The regulation of non-combustible nicotine delivery products and cigarettes should be proportionate to their relative risks, so that smokers have incentives to switch from combustibles to safer alternatives, and cigarette companies have incentives to promote safer products.

## Introduction

The main reason people use tobacco is to obtain nicotine, the psychoactive and addictive substance that, among other effects, both stimulates and calms the body.^1^ The brain effects of nicotine actions are complex but show powerful reinforcing effects via the neural dopaminergic system, which is central in the neurobiology of addiction.^2^ Not all tobacco products deliver nicotine in the same way.^3^ The invention of flue-cured tobacco and its introduction into cigarettes helped make cigarette smoke more inhalable and thus highly addictive, making cigarettes the leading form of tobacco consumed worldwide since the early part of the 20th century.^3,4^ When cigarette smoke is inhaled, nicotine travels quickly to the lungs, arterial blood, and the brain where it exerts addictive effects.^2^ Cigarettes and their variants, such as roll-your-own cigarettes, pose the highest risks for disease because their design allows for mildly acidic smoke to be readily inhaled into the lungs with less discomfort than the more alkaline smoke found in most pipes and cigars.

From the middle part of the 20th century, public health advocates were locked in battle with an oligopolistic tobacco industry that denied health risks and circumvented regulation, even as evidence showing the link between smoking and disease became undeniable.^4,5^ However, this Manichean struggle^6^ was disrupted in the early part of the 21st century by product innovation which allowed a growing spectrum of lower-risk nicotine delivery products to reach consumers, threatening to displace cigarettes as the dominant form of nicotine delivery. Nicotine vaping products (NVPs), also referred to as electronic cigarettes or e-cigarettes, began to be sold online by Chinese firms, although their popularity in the United States did not grow substantially until 2012.^7^

To the extent that NVPs are less harmful than cigarettes, they yield public health gains when used by never-smokers who otherwise would have initiated smoking or used by smokers who otherwise would not have quit smoking.^8^ In contrast, vaping increases harm when used by never-smokers who otherwise would not have started smoking or by smokers who otherwise would have soon quit smoking.^8^ Since cigarettes are a longstanding and highly profitable source of revenue,^9–11^ cigarette companies generally have incentives to follow the latter path—that is, encouraging vaping only as a gateway to smoking or to maintain smoking instead of quitting—unless there are financial imperatives to promote potentially harm-reducing products.

At least one cigarette company, Philip Morris International (PMI) has claimed that it sees the company’s future in “harm reduction” products rather than cigarettes.^12^ Altria (formerly part of Philip Morris and now separate from PMI) states, “We’re building a diversified business model with smoke-free products to further our harm reduction goals and achieve our Vision by 2030 to responsibly lead the transition of adult smokers to a smoke-free future.”^13^ Other cigarette companies have also diversified into non-combustible tobacco products, although they have been less explicit about their intention to change.^14–16^

The purpose of this study is to examine the financial incentives of cigarette companies to encourage the use of non-combustible nicotine delivery products (NCNDPs) as a substitute for cigarettes. We analyze their incentives from a profit-maximizing point of view, whereby firms are willing to forgo current profits from cigarettes to obtain future profits from NCNDPs. We identify the conditions under which cigarette companies are more likely to follow through on their stated commitments to selling NCNDPs. At the same time, we recognize the difference between public health goals from the profit-maximizing goals of private companies. Our study focuses on the behavior of cigarette companies in the United States, where the issues surrounding competition and market structure are prominent and the evidence is most abundant. However, we expect that similar issues arise in other countries.

## Methods

In light of the limited and heterogeneous literature on market structure and competition, we provide a narrative review of the relevant literature. Our study reviews previous literature drawing largely on previous studies by Levy et al.^17–21^ that focus specifically on the role of competition and market structure. To update our previous review articles,^16,19^ we conducted a search of citations of those articles using Google Scholar and conducted a search of PubMed and EconLit for other more recent relevant literature. We used search terms as described in our previous review articles^16,19^ including “industry,” “market,” and “competition” paired with “cigarettes,” “e-cigarettes,” “ENDS,” “NVPs,” “heated tobacco products,” and “oral nicotine pouches.” The search was completed in January 2022.

Our paper also includes original empirical research. To examine the impact of NVP use on competition from cigarette companies, we examined the relationship between the growth in NVP use and both smoking prevalence and cigarette company profits. Specifically, we considered how changes in smoking prevalence trends and stock market price trends correspond with the increased use of NVPs. Applying a trend-line analysis to cigarette prevalence rates from the National Health Interview Survey, we tested for changes in the trend that correspond to increased NVP use. We also examined changes in the stock market prices of cigarette firms relative to an overall financial market index, focusing on the years in which NVP sales by noncigarette companies showed rapid growth.

## Results

### Market Structure and Competition

To distinguish the changing role of market structure and competition, we consider three separate time periods. Since the market position of cigarette companies began to undergo fundamental changes around 2006, we first consider competition in the period before 2006 when the companies focused almost exclusively on selling cigarettes. While there is not a clear demarcation of when market competition changed, we chose the year 2006 because at about that time, consumers and cigarette companies began showing greater interest in other nicotine delivery products. We distinguish the time after 2006 into two periods. From 2006 through 2012, cigarette smokers, especially the young, increasingly used other tobacco products, especially smokeless tobacco. In addition, cigarette companies bought up firms producing other tobacco products to maintain market power. While the period 2006–2012 demonstrated the growing interest of cigarette firms in other tobacco products, the period since 2012 corresponds to the growth of an alternative (nontobacco) nicotine delivery product, NVPs. The period since 2012 can be characterized as a period of rapid growth in NCNDPs, most notably NVPs, with cigarette firms facing potent competition from noncigarette companies selling those products.

### Competition in the Pre-2006 Period

A large pre-2006 economics and marketing literature^17,21^ on cigarette industry competition as well as antitrust cases brought by the Federal Trade Commission^22–24^ positioned cigarette firms as a stand-alone industry, due to the lack of close substitutes for cigarettes from the perspective of both consumers and companies.^17,21^ In maximizing profits, firms in that industry had market power whereby they could raise prices independently of firms producing other products, including other tobacco products such as smokeless tobacco.^17,21^

Economics studies have generally found that the pricing behavior of U.S. cigarette companies could be characterized as anti-competitive.^17,21^ While evidence of explicit collusion is limited, studies indicate that the major cigarette companies acted in a way that could be characterized as implicit collusion with dominant firm pricing, whereby pricing is coordinated to maximize their overall profits.^17,21^ This behavior is consistent with the market structure of the U.S. cigarette industry. That market has grown highly concentrated, with Altria now having a market share of over 50%, and, along with British American Tobacco (BAT) and Imperial, the major cigarette companies have over 90% of the market by 2004. Because of the small ­number of larger firms controlling most of the market, they could coordinate their behavior.^17,21^

The ability of the major existing firms to control the distribution of cigarettes while maintaining higher prices and profits depends on limiting the growth of smaller rival firms and preventing new firms from taking a major role. Barriers to the growth of smaller rivals and the entry of new firms include: the importance of advertising at scale and creating brand recognition (e.g. the Marlboro brand name); product proliferation to discourage rival firms from exploiting market niches; retail slotting allowances (i.e. fees paid by producers to have retail firm display their products); and legal barriers, such as the threat of lawsuits and the lack of financial and technical resources to comply with tobacco control regulations.^17,21^ With most tobacco sales taking place in conventional retail, slotting allowances are particularly important as they enable cigarette companies to buy up limited retail shelf space, thereby keeping potential entrants or fringe firms from gaining sufficient market share to cover profits.^25,26^ The major firms were able to supplement these entry barriers with predatory pricing (e.g. Marlboro Friday^27^), in which firms temporarily reduce prices to deter entry or limit rivals. Price discrimination, in which firms selectively reduce prices to key vulnerable customers, such as youth and low socioeconomic status smokers, has also enabled firms to limit entry and retain their most important customers.^17,21^

In summary, prior to 2006, cigarette companies specialized almost exclusively in cigarettes, which were distributed almost exclusively through conventional retail. Other tobacco products, such as smokeless tobacco and cigars, were largely separate markets. With a small number of major firms and facing limited competition, the major cigarette companies were able to act in unison to maintain their market power.

### Diversification From 2006 Through 2012

During 2006–2012, firms were able to maintain much of their market power. However, from 2006, tobacco products other than cigarettes gained more interest among consumers primarily because of their convenience (use of non-combustible products indoors) and the lower cost relative to cigarettes. While U.S. cigarette sales continued their earlier decline, smokeless tobacco sales, mostly snuff, increased through 2013.^28^ Consumers, especially youth and young adults,^29,30^ increasingly became poly-tobacco users,^31,32^ often using cigarettes alongside other tobacco products.^33^ In particular, dual use of smokeless tobacco and cigarettes became more common^34^ and little cigar sales grew rapidly.^35–37^

Cigarette companies clearly recognized the impact of other tobacco product use on their profitability. Reynolds American acquired Conwood Smokeless Tobacco Company in 2006, Altria acquired the U.S. Smokeless Tobacco Company in 2009, and both companies introduced brand extensions, for example, Camel Snus and Marlboro Snus.^17,21,38^ With the acquisition of the largest smokeless companies and the brand loyalty of their customers, plus brand extensions to new products and the ability to restrict entry through slotting allowances, the cigarette industry became dominant in the smokeless tobacco market. In response to smoke-free laws, cigarette companies marketed smokeless tobacco as a way for smokers to satisfy their nicotine cravings when smoking was restricted in the workplace.^39,40^ In 2007, Altria purchased the cigar company John Middleton Company, makers of the popular Black & Mild mass-market cigar, further expanding their control of other tobacco products.^41^

### The Post-2012 Years and the Increasing Role of NVPs

Although the sales of almost all tobacco products fell during 2013–2018, alternative nicotine delivery products, notably NVPs, began to show rapid sales growth.^17–21^ NVPs were available in the United States by 2009, but their use was minimal until 2012 when a new generation of products became available that more efficiently delivered nicotine.^13,42,43^ NVP use increased,^44,45^ especially between 2011 and 2013 and later in 2017.^46^ By 2017, four generations of products had been developed, each delivering nicotine more efficiently than the last, thereby providing a more effective substitute for cigarettes. Unlike smokers, vapers had access to a wide variety of device types, flavors, and nicotine strengths,^19,21^ and NVPs could be purchased over the internet and from specialist vape shops as well as through conventional retail.^19,21^

Although studies prior to 2012 indicated that cigarette use was relatively unresponsive to price,^47^ studies since 2012 find that cigarette consumption is much more responsive to price (i.e. price elasticities are greater than one), indicating greater substitutability by consumers switching to other products.^42,48–50^ Recent studies also explicitly find that NVPs,^43,48–50^ cigars,^36,37^ and smokeless tobacco^48^ are relatively close substitutes for cigarettes. Thus, recent demand studies indicate the importance of NVPs to cigarette sales and more broadly the competition between nicotine delivery products.

Unlike for cigarettes, cigars, and smokeless tobacco products, where brand name is important and products are mostly sold through conventional retail, the production and marketing process for NVPs are consistent with ease of entry by new firms.^19,21^ In manufacturing vaping devices, ­technological barriers from economies of scale or proprietary knowledge are minimal. Vaping devices are relatively straightforward to produce and nicotine liquids can even be mixed by consumers in their homes.^19,21,44^ Many firms contract with outside companies, particularly those based in China, for the manufacture of their products.^19,21^ Much of the marketing takes place over the internet,^19,21,44,45^ especially through social media and by word of mouth. More costly forms of advertising, such as mass broadcast media, are not essential.^19,44,46^ Since most vaping sales have been through the internet and vaping shops, retail slotting fees do not pose a significant entry barrier.^19,21^ Prior to 2020, government regulations placed few limits on NVPs and did not pose a significant deterrent to entry into the United States.

Compared to the high concentration and market share stability in tobacco markets, many different firms were selling vaping products. The products were initially manufactured and marketed by start-up companies outside the cigarette industry (“independents”), such as NJOY. The 2016 Surgeon General’s Report on NVPs^2^ lists three primary groups of 33 different vaping device sellers in the 2015 U.S. market: (1) cigarette companies selling NVPs, (2) independent public companies, and (3) independent, privately owned NVP companies.^2^ As shown in Table 1, there was considerable instability in conventional retail market share, with rapid turnover of the leading firms.^17,19,51^ For example, a previous leader, NJOY, filed for bankruptcy in 2016, but later came back with strong sales.^19,21,51^

**Table 1.** Nicotine Vaping Product Market Shares of the Three Leading Firms in Conventional Retail, by Year.* Cowen Reports

Company/Year	2011	2012	2013	2014	2015	2016	2017	2018
Ballantyne	17%							
CB **distributors**	23%	24%						
NJOY	30%	22%	22%					
Imperial		17%	42%	33%	22%	28%		
Japan **Tobacco International**			12%	16%	14%			
British American Tobacco (Reynolds)				15%	33%	38%	30%	21%
Altria						14%	15%	12%
**JUUL**							25%	55%

^*^Cowen reports.^51^ These figures exclude specialist vape shops and internet sales. Only included is conventional retail, which is estimated to make up less than 30% of total sales.

Starting in 2012, major cigarette companies entered the vaping market, with Lorillard acquiring Blu, Japan Tobacco International acquiring Logic, and with Altria introducing MarkTen.^19,21^ In late 2013, Reynolds brought Vuse onto the conventional retail market with aggressive advertising and price discounts.^44^ By the end of 2014, Vuse had become the market leader in conventional retail, with its share reaching 36% in late 2015.^52^ Altria began marketing MarkTen in 2014, and its conventional retail market share reached 16% by the end of 2015.^52^

Because of the past dominance of the major cigarette companies in controlling the marketplace, many public health advocates^53–56^ have implied or assumed that the cigarette industry was the major force behind the growth of the NVP market. However, this perception is not accurate. Although cigarette companies gained a relatively large share of NVP sales in conventional retail by September 2017,^19,21,57^ the combined market share of all cigarette companies in the overall market (including vape shops and the internet) at its height was likely below 25% (with conventional retail at about 30% of the overall market).^19,21^ In addition, there was considerable instability in market shares.^19,21^ Indeed, cigarette companies quickly lost their dominance in conventional retail.

The cigarette companies’ overall share of vaping sales within conventional retail was at its height in 2017. However, Pax Labs, an independent, entered the market in June 2015 with JUUL replacing Vuse as the market leader by the end of 2017. JUUL grew from 9% of conventional retail dollar sales in June 2017 to 77% by December 2018.^58^ After the introduction of JUUL, the conventional retail share of all cigarette firms fell from 78% in September 2017 to 25% by July 2018.^19,57,59^ JUUL’s market share in the overall vaping market was estimated at 30%,^60^ while those of cigarette companies totaled less than 10%. A 2019 estimate put the combined global share of NVPs by cigarette companies at 17.6%.^61^ After December 2018, JUUL’s growth tapered off and it soon faced both the tobacco industry and independent competitors producing similar “pod mod” products (e.g. Imperial’s Myblu, BATs Vuse Alto, Suorin Air). These in turn were challenged by the rapid growth of disposable NVPs.^62^

Other recent developments also reveal the importance of NCNDPs to cigarette companies. In late 2018, Altria purchased a major stake in JUUL at a surprisingly high price,^18,20,21,60^ indicating the importance to Altria of gaining greater control of the overall nicotine delivery market.^7^ In May 2019, Altria was granted permission to market IQOS, a PMI-heated tobacco product with a substantial market share in Japan.^63^ Like vaping products, heated tobacco products (HTPs) are inhaled and provide a similar sensorimotor experience and “throat-hit” to cigarettes. In the United States, British American Tobacco not only attempted to enter the U.S. market but sued Altria for patent infringement leading to their removal from the market.^64^ In 2019, Altria purchased a majority interest in On!,^65^ an oral nicotine delivery product.

In sum, the market landscape has markedly shifted from a focus on cigarettes to what might be described as a broader nicotine delivery product market, which includes cigarettes, cigars, smokeless tobacco (including new oral nicotine products), HTPs and NVPs. Competition has increased because of the relatively easy entry of new players, whose successes in the NVP market spurred cigarette companies to replicate or buy into their products.

### The Impact of NVPs on Cigarette Companies from a Profit-Maximizing Perspective

In the previous section on the post-2012 years, we argued that consumers and cigarette companies have shown interest in NCNDPs, most notably NVPs. In this section, we consider direct incentives for cigarette companies to sell NVPs. These incentives depend on the trade-off between the high profits from continuing to focus on selling cigarettes versus the ­profits from replacing cigarettes with NCNDPs. From a profit-maximizing perspective, the importance of NCNDPs to cigarette companies is supported by evidence of: (1) changes in smoking and vaping prevalence, and (2) the impact of vaping growth on cigarette stock prices.

**Figure 1.**
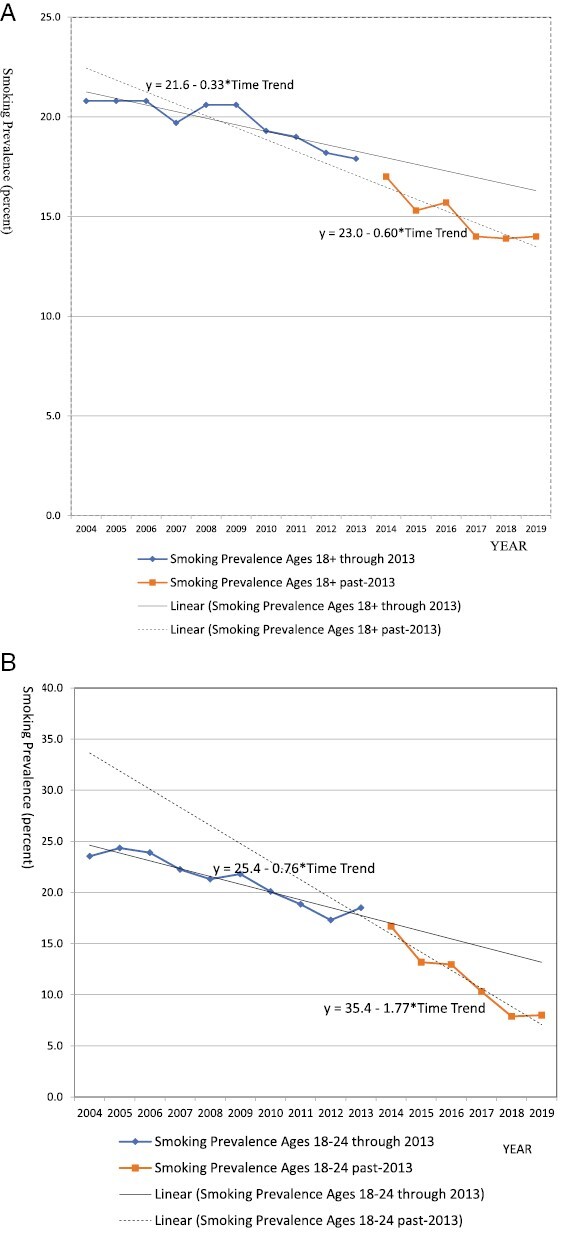
(A) U.S. smoking prevalence, ages 18 years and above, national health interview survey, 2004–2012 and 2013–2019. (B) U.S. smoking prevalence, ages 18–24 years, national health interview survey, 2004–2012 and 2013–2019.

Recent studies and industry reports indicate that smoking prevalence has fallen at a much greater rate since NVP use became more widespread in 2012.^7^ As shown in Figure 1A, data from the U.S. National Health Interview Survey^66^ indicate that adult (males and females ages ≥18) smoking prevalence fell at an average rate of 0.3 percentage points per year from 2004 to 2012 (as indicated by the slope of the linear trend line for the pre-2013 period), then fell twice as fast by 0.6 percentage points per year from 2013 to 2019 (as indicated by the slope of the linear trend line for the post-2013 period). Levy et al.^67^ applied an indirect method, comparing survey smoking rates to counterfactual smoking rates projected without NVPs but incorporating policy changes. They estimated net NVP-related relative reductions in U.S. adult smoking prevalence of 10%–13% over 2013–2018. Using that same method for England, Levy et al.^68^ estimated NVP-related relative reductions in adult smoking prevalence of about 14%–20% over 2012–2019. Thus, recent trends indicate a rapid acceleration of the decline in smoking prevalence that coincides with growing NVP use.

Although current profits tend to reflect current adult smoking prevalence, future profits depend more on sales to current youth and young adults. These groups make up future ­generations of smokers and are among the highest NVP users.^7^ As shown in Figure 1B.,^66^ ages smoking prevalence among 18–24 year olds in the United States fell at an average annual rate of 0.8 percentage points from 2004 to 2012 and 1.7 percentage points from 2013 to 2019. Two recent studies^69,70^ also found more than twice the reduction in youth smoking prevalence in 2013–2018 compared to prior years. Levy et al.^67^ estimated net NVP-related U.S. smoking prevalence reductions of 43%–53% for ages 18–24 over 2013–2018. Continued reductions in young adult smoking will accelerate declining smoking prevalence and cigarette sales into the future in much the same way that a population is sure to eventually fade away if it fails to reproduce (i.e. to replace the adult smokers who quit and/or die each year). These reductions suggest declining profits from cigarette sales will continue in the future if NCNDP use continues to grow.

Stock market prices provide a direct measure of the anticipated profitability of cigarette companies, and thus an even more direct gauge of the impact of growth in the NCNDP market. A firm’s stock price relative to an overall financial market index is regarded in the finance literature^71–74^ as a reflection of investors’ best evaluation of current and (discounted) future profitability at a particular point in time, and as such is a central concern of companies owned by their stockholders. Examining trends in stock prices is also a method used by tobacco control researchers^75–78^ to gauge the impact of an event on profitability and avoids the problems typically associated with developing accurate estimates of current and future profits.^9,17,21,79^

Although cigarette companies are traditionally considered a good investment,^80^ stock prices in recent years have directly reflected the major challenges that U.S. cigarette companies have faced from NVPs. We examine changes in stock price trends at critical points in the growth of NVP sales, particularly during the rapid growth of independent (non-tobacco industry) firms. Smoking prevalence, especially that of youth and young adults, began its rapid decline in about 2014, signaling the loss of future profits. However, by this time, cigarette companies had successfully entered the NVP market. In 2016, the deeming rule of the U.S. Dood and Drug Administration (FDA) extended their jurisdiction to NVPs.^81^ In 2017, the FDA announced its intent to encourage the use of less hazardous nicotine delivery products as alternatives to combustible tobacco products.^82^ In addition, JUUL began its rapid sales growth, greatly surpassing the sales of cigarette company NVP products. By 2017, it was clear that NVPs were here to stay, and that non-cigarette companies would have a major stake in these products.

**Figure 2.**
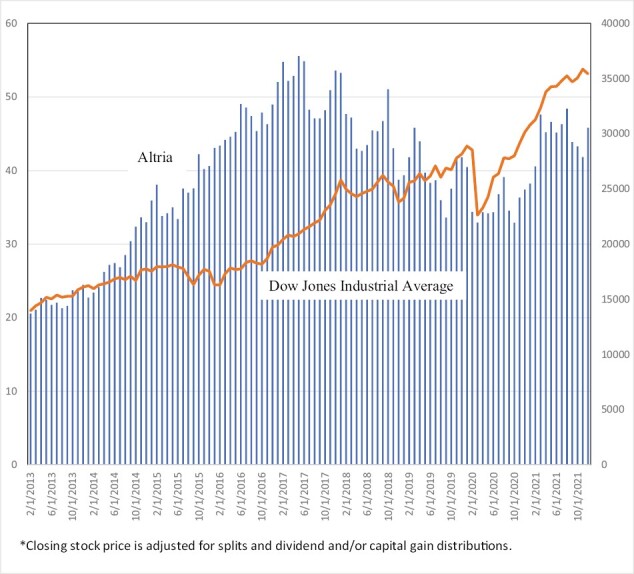
Stock prices:* Altria versus Dow Jones Industrial Average, 2013–2021.

**Figure 3.**
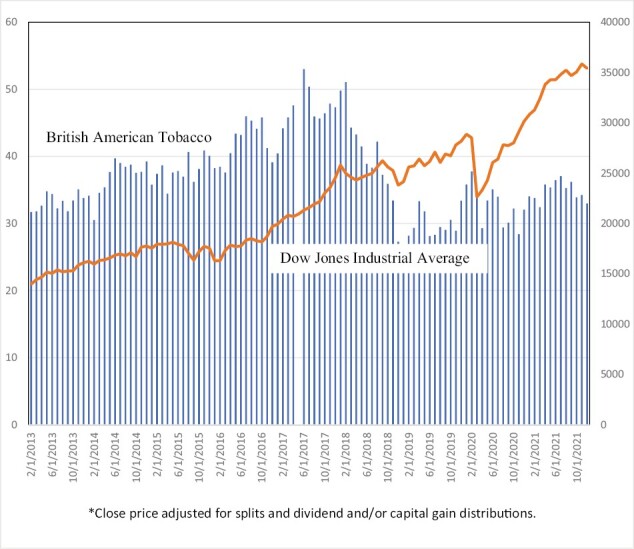
Stock prices*: British American Tobacco versus Dow Jones Industrial Average, 2013–2021. *Close price is adjusted for splits and dividend and/or capital gain distributions.

Figures 2 and 3 show stock prices from 2013 to 2021 of Altria and BAT in comparison to the Dow Jones Industrial Average, a general market-wide stock index. Altria’s stock price (adjusted to reflect dividends) did well relative to the Dow Jones Industrial Average before 2017, but then fell from $56/share in early 2017 to $34/share by 2019, a 40% drop. British American Tobacco’s adjusted stock price increased with the market before 2017, but its stock price fell from $53/share to $25/share from late 2016 to early 2019, a 56% drop. Over a roughly corresponding time period, the Dow Jones Industrial Average grew by over 30%, indicating that cigarette stock prices declined despite solid growth in the overall stock prices. As shown in Supplementary Figures 1 and 2, Imperial Brands and Phillip Morris International experienced similar patterns.^83^

Following the initial drop in 2020, the recovery in cigarette stock prices in early 2020 may be explained at least in part by FDA announcements and policies giving cigarette companies advantages in selling NCNDPs. Under the FDA’s 2016 deeming rule,^84^ new and existing vaping products require approval prior to market entry and for market continuation. However, the FDA had not been enforcing the premarket tobacco product applications (PMTA) pathway until announcements were made in late 2019 requiring that applications for deemed products were to be received by May 20, 2020.^85^ By December 20, 2021, the FDA had denied the approval of over one million vaping product applications from smaller companies, with over 5 million “refuse to file” determinations and only one approval given to BAT’s Vuse Solo tobacco-flavored product.^86^ Both Altria’s and British American Tobacco’s stock prices increased slightly from early 2020. PMI and Swedish Match have seen more rapid growth in their stock prices since early 2020, coinciding with success in their FDA applications for IQOS and oral tobacco products and their global success in marketing NCNDPs (See Supplementary Figures 2–3).^86,87^

### Future Incentives of Cigarette Companies

Based on the experience of the last 10 years in the United States, cigarette companies will develop and promote NCNDPs only to the extent that they feel compelled to do so by competition. To the extent that future profits depend on switching customers to NVPs, cigarette companies will likely seek to promote those NCNDPs which will be most profitable over time.^19,21^ Government regulations will play an important role.

Although Altria acquired a large share of JUUL in December 2018, the merger is currently being challenged by the Federal Trade Commission and, further, it is not clear that Altria will promote Juul over its other products. Cigarette companies may continue to maintain a presence in the NVP market, but they have greater incentives to more actively promote HTPs instead of NVPs. HTPs, such as IQOS, provide the potential for greater profitability because of the more proprietary nature of the technology.^19,21^ Based on what has happened in other countries,^88,89^ cigarette companies are unlikely to face competition from non-cigarette firms in this submarket. Consequently, the HTP submarket is likely to be highly concentrated in the same way as current cigarette markets. In 2018, PMI was reported to have almost 80% of the global HTP market, but just 0.3% of the global e-cigarette market.^90^

Modern oral nicotine pouches are another potential substitute for combusted tobacco. From the cigarette companies’ perspective, products that allow consumers to top up their nicotine levels even in smoke-free environments^47^ have the potential to reduce smoking cessation, thus protecting company profits from combusted cigarettes. In addition, modern oral nicotine pouches provide another way of expanding the overall nicotine delivery market.^24,45,46,51,52^ Cigarette company-owned nicotine delivery products, On! by Altria^65^ and Velo by BAT^91^ face competition among themselves as well as from Swedish Match, a company specializing in oral nicotine products including the top-selling MONP Zyn.^92^ In May 2022, PMI offered to acquire Swedish Match,^93^ potentially providing PMI direct access to the U.S. market with control of Swedish Match’s infrastructure and oral products, most notably Zyn. Recently, PMI also purchased pharmaceutical inhaled nicotine product, Vectura.^94^ By offering a broad array of nicotine delivery products, cigarette companies can target different consumer tastes, thereby refining marketing efforts to expand sales with stronger market segmentation. The sale of NCNDPs also allows cigarette companies to maintain and potentially expand their customer base to include not just smokers, but also aspiring and actual quitters alongside new users of a variety of NCNDPs.^8,95^

The incentive for cigarette companies to actively promote NCNDPs and de-emphasize conventional tobacco products will depend on market regulation, and specifically how regulations affect competition. As described above, firms were not subject to the FDA’s PMTA approval process until 2021.^96^ Industry analysts had predicted that the deeming rule would favor major cigarette companies “because of their larger financial resources and regulatory experience.”^97,98^ Experience to date indicates that cigarette companies will be most successful in gaining approval of PMTAs.^99^ Unless independent companies are also approved, cigarette companies will face less competition and have less incentive to promote NCNDPs over cigarettes.^19,21^ While PMTAs are meant to protect against the use of harmful products, especially by the young, regulations such as these can act as a barrier to entry that reduces competition, thereby lessening the incentives ­for ­cigarette companies to invest in NCNDPs. Whilst the transformation of the tobacco industry to favor NCNDPs could conceivably occur in the absence of competition from independent firms, it would certainly be hastened by the threat of losing customers to independent firms.

Tobacco control policies may also play a major role in any transformation of the cigarette industry.^100^ Regulations that make NVPs less appealing (e.g. flavor bans, taxes leading to price increases) will likely slow the transition of smokers to NVP products. Conversely, regulations that reduce consumer information problems, such as prohibiting toxic ingredients and providing accurate information about the risks of NCNDPs relative to cigarettes, are likely to encourage smokers to switch to these products and incentivize cigarette companies to promote them. In addition, state and/or federal regulations of nicotine levels can reduce the addictiveness of particular brands. Perhaps even more important are policies that reduce the appeal of cigarettes, for instance, tax increases^100,101^ and a ban on menthol in cigarettes.^102^ As argued above, demand studies indicate that NVPs have become a close substitute for cigarettes,^43,48–50^ so policies making cigarettes less desirable can also have an important impact. Indeed, with NVPs and HTPs as substitutes for cigarettes, the impact of these policies may be greater than in previous decades.

Although cigarette companies are likely to strategically promote NCNDPs, they will do so in reaction to ­competition either from independent firms or from other cigarette firms attempting to gain a strong foothold in the market. Cigarettes are highly profitable^9–11^ and if their market share is not eroded by smokers switching to other products, cigarette companies’ profits are likely maximized by continuing to sell cigarettes. For example, while cigarette companies reduced both cigarette and smokeless tobacco marketing expenditures between 2013 and 2019, they continued to concentrate the remaining expenditures on discounting prices to the most vulnerable groups, such as low socio-economic status and high-intensity smokers.^103^ Rather than raise prices to these price-sensitive groups, they have instead discounted prices, the likely result of which is to maintain these groups as cigarette customers.^104^

The incentives to promote NCNDPs and place less emphasis on cigarettes may also differ by company. British American Tobacco, which in the United States mainly sells menthol cigarettes through its Reynolds subsidiary, recently challenged the FDA’s attempts to ban menthol in cigarettes.^105^ As indicated above,^12–16^ cigarette companies have expressed varying degrees of support for replacing cigarettes with NCNDPs. Furthermore, while we have focused on the United States, cigarette companies continue to maintain a strong presence and promote cigarette use in low- and middle-income countries,^106,107^ where sales of NVPs are often restricted or even banned.

In sum, a transformation of U.S. cigarette companies towards a new focus on NCNDPs will continue to depend on market competition and particularly government regulatory policy. Stronger combusted tobacco control policies, such as higher cigarette taxes, can encourage smokers to quit all nicotine products or at least switch to less harmful products. Conversely, regulations that limit competition from independent firms, but protect cigarette firms’ profits risk slowing or even reversing the recent large declines in smoking, especially among youth and young adults. Policies directed towards NCNDPs and cigarettes should be proportionate to their health risks,^108^ so that smokers have incentives to continue to switch from combustibles to safer alternatives, and cigarette companies have incentives to promote these less harmful products. Tobacco control policy should not be focused primarily on the highly concentrated cigarette market. In the various fragmented submarkets of nicotine delivery products, tobacco control policies and regulations should be balanced across products and reflect the risks associated with each of the different products.^100,109^

## Conclusions

With the emergence of NVPs, a highly concentrated cigarette market has evolved into a highly fragmented nicotine delivery product market. In the period before the growth of NVPs and the nicotine delivery product market became competitive, tobacco markets were stable and the interests of the cigarette companies were clear. Cigarette companies focused on cigarettes and maintained their profits through pricing and marketing strategies and by opposing measures likely to reduce cigarette sales. When the nicotine delivery product landscape started evolving in about 2006, cigarette companies quickly reacted by diversifying into smokeless tobacco, cigars, and NVPs. When NVPs became more widely used in about 2013, cigarette companies began to offer these products, but they have not achieved the same oligopolistic control that they have enjoyed for cigarettes. In this paper, we have argued that U.S. cigarette companies will back alternatives to cigarettes when threatened by competition, but likely those with the greatest profit potential, which may mean HTPs rather than NVPs.

We are a long way from the narrative of transformation put forward by some cigarette companies becoming a reality. As such, a skeptical view of cigarette companies is warranted, and it will be important that regulatory regimes around NVPs help rather than hinder that transformation. The prospects for a lasting conversion of cigarette companies to NCNDPs are likely to depend at least in part on the degree of competition in the various nicotine delivery submarkets, itself largely a function of government regulation in this area. The incentives for cigarette companies to promote NCNDPs instead of cigarettes depend, in part, on the pressure that they face from non-cigarette companies. In the absence of that competition, those companies have less incentive to promote NCNDPs. If government regulations have the effect of reducing competition, we can also expect less innovation in the shape of less harmful nicotine delivery product alternatives and better substitutes for cigarettes. At the same time, public health advocates and researchers need to be open to the concept of both cigarette and non-cigarette companies earning profits from selling NCNDPs that will likely cause dependence in some users while at the same time offering consumers alternatives to combusted products.

Our study is subject to limitations. Our work is largely drawn from previous work of the authors. While that work involved reviews that we have updated for the current study, additional research is needed to study industry dynamics and to examine the impact of regulations on tobacco industry-owned versus independent NCNDP companies. In addition, we focused on the United States. Research is needed for other countries. Industry analyses should be an ongoing process because of the rapid innovation of new products and changes in the role of industry and government.

Tobacco control researchers have a key role to play in analyzing the impact of government regulations on competition in the nicotine delivery product market and on the market position of NCNDPs relative to cigarettes. Regulations that weaken independent companies and competition open the way for large cigarette companies to dominate the NCNDP market with whatever product is most profitable and least likely to damage their combustible product business. At the same time, as profit-maximizing firms, cigarette companies have incentives to expand the base of these products to new users of nicotine delivery products as well as those who would have otherwise smoked cigarettes. We must also continue to expose tobacco company activities that promote or maintain smoking and discourage the use of NCNDPs, such as continued marketing expenditures to discount cigarette prices, lobbying or litigating for regulations that protect cigarette profitability, and continued investment in combustibles throughout the world.

## Supplementary Material

A Contributorship Form detailing each author’s specific involvement with this content, as well as any supplementary data, are available online at https://academic.oup.com/ntr.

ntad014_suppl_Supplementary_Figures

## Data Availability

All data used in the current study will be provided on request.

## References

[1] U.S. Department of Health and Human Services. Nicotine Addiction. A Report of the Surgeon General, 1988. Atlanta, GA: U.S. Department of Health and Human Services, Centers for Disease Control and Prevention, National Center for Chronic Disease Prevention and Health Promotion, Office on Smoking and Health; 1988.

[2] Prochaska JJ , Benowitz NL. Current advances in research in treatment and recovery: Nicotine addiction. Sci Adv. 2019;5(10):eaay9763. 31663029 10.1126/sciadv.aay9763 PMC6795520

[3] Slade J. Nicotine delivery devices. In: Orleans CT, Slade J, eds. Nicotine Addiction: Principles and Management. New York: Oxford University Press; 1993.

[4] Brandt A. The Cigarette Century: The Rise, Fall, and Deadly Persistence of the Product That Defined America. New York: Basic Books; 2007.

[5] Proctor R. Golden Holocaust: Origins of the Cigarette Catastrophe and the Case for Abolition. Berkeley: University of California Press; 2011.

[6] Borland R , Young D, Coghill K, Zhang JY. The tobacco use management system: analyzing tobacco control from a systems perspective. Am J Public Health. 2010;100(7):1229–1236. 20466970 10.2105/AJPH.2009.165910 PMC2882395

[7] Levy DT , Yuan Z, Li Y, Mays D, Sanchez-Romero LM. An examination of the variation in estimates of e-cigarette prevalence among U.S. adults. Int J Environ Res Pub Health. 2019;16(17):1–19. 10.3390/ijerph16173164 PMC6747488 31480240

[8] Levy DT , Cummings KM, Villanti AC, et al. A framework for evaluating the public health impact of e-cigarettes and other vaporized nicotine products. Addiction. 2017;112(1):8–17. 27109256 10.1111/add.13394 PMC5079857

[9] Branston JR. Industry profits continue to drive the tobacco epidemic: a new endgame for tobacco control? Tob Prev Cessat. 2021;7(June):45. 34179591 10.18332/tpc/138232 PMC8193577

[10] Gilmore AB , Branston JR, Sweanor D. The case for OFSMOKE: how tobacco price regulation is needed to promote the health of markets, government revenue and the public. Tob Control. 2010;19(5):423–430. 20876078 10.1136/tc.2009.034470 PMC2981493

[11] American Cancer Society. New Tobacco Atlas Estimates US $35 billion tobacco industry profits and almost 6 million deaths. http://pressroom.cancer.org/releases?item=356. Published 2012. Accessed December 8, 2021.

[12] Philip Morris International. Our transformation. https://www.pmi.com/our-transformation. Published 2021. Accessed December 4, 2021.

[13] Altria. Our Vision. http://altria.com/about-altria/our-vision. Published 2021. Accessed December 10, 2021.

[14] British American Tobacco. Understanding the comparative risks of our products. https://www.bat.com/harmreduction. Published 2021. Accessed December 10, 2021.

[15] JTI. Reduced-Risk Products – Our Vaping Products. https://www.jti.com/about-us/what-we-do/our-reduced-risk-products. Published 2021. Accessed December 10, 2021.

[16] Imperial. Next Generation Products. https://www.imperialbrandsplc.com/about-us/next-generation-products.html. Published 2021. Accessed December 10, 2021.

[17] Levy D , Chaloupka F, Lindblom E, et al. The US cigarette industry: an economic and marketing perspective. Tob Reg Sci. 2019;5(2):156–168. 10.18001/trs.5.2.7 PMC7454012 32864394

[18] Levy D , Douglas C, Sanchez-Romero L, Cummings K, Sweanor DT. An analysis of the FTC’s attempt to stop the altria-juul labs deal. Tob Reg Sci. 2020;6(4):302–305. 10.18001/TRS.6.4.7 PMC7475750 32901222

[19] Levy D , Lindblom E, Sweanor D, et al. An economic analysis of the pre-deeming US market for nicotine vaping products. Tob Reg Sci. 2019;5(2):169–181. 10.18001/trs.5.2.8 PMC7454013 32864395

[20] Levy DT , Sweanor D, Sanchez-Romero LM, O'Connor R, Goneiwicz M, Borland R. Altria-Juul Labs deal: why did it occur and what does it mean for the US nicotine delivery product market. Tob Control. 2020;29(e1):e171–e174. 31484802 10.1136/tobaccocontrol-2019-055081 PMC7279136

[21] Levy D , Douglas C, Sanchez-Romero L-M, Cummings M, Sweanor D. An antitrust analysis of the JUUL-Altria deal: antitrust and population health implications. JCLE. 2021;17(2):458–492. 10.1093/joclec/nhaa033 PMC8318606 34335858

[22] United States v. American Tobacco Co, 221 106(Supreme Court 1911).

[23] American Tobacco Co. v. United States. 1946;328 U.S. 781(No. 18).

[24] Federal Trade Commission. RJ Reynolds Tobacco Holdings, Inc./British American Tobacco. p.l.c. US: Federal Trade Commission; 2003.

[25] Kaikati JG , Kaikati AM. Slotting and promotional allowances: red flags in the supply chain. Supply Chain Manag:Int J. 2006;11(2):140–147.

[26] Marx LM , Shaffer G. Slotting allowances and scarce shelf space. J Econ Manage Strategy. 2010;19(3):575–603.

[27] Chen T , Sun B, Singh V. An empirical investigation of the dynamic effect of Marlboro’s permanent pricing shift. Marketing Sci. 2009;28(4):740–758.

[28] Wang TW , Kenemer B, Tynan MA, Singh T, King B. Consumption of combustible and smokeless tobacco - United States, 2000-2015. MMWR Morb Mortal Wkly Rep. 2016;65(48):1357–1363. 27932780 10.15585/mmwr.mm6548a1 PMC5584068

[29] Bombard JM , Rock VJ, Pederson LL, Asman KJ. Monitoring polytobacco use among adolescents: do cigarette smokers use other forms of tobacco? Nicotine Tob Res. 2008;10(11):1581–1589. 18988070 10.1080/14622200802412887

[30] Rath JM , Villanti AC, Abrams DB, Vallone DM. Patterns of tobacco use and dual use in US young adults: the missing link between youth prevention and adult cessation. J Environ Public Health. 2012;2012:679134. 22666279 10.1155/2012/679134 PMC3361253

[31] Sung HY , Wang Y, Yao T, Lightwood J, Max W. Polytobacco use of cigarettes, cigars, chewing tobacco, and snuff among US adults. Nicotine Tob Res. 2016;18(5):817–826. 26136525 10.1093/ntr/ntv147 PMC5896811

[32] Lee YO , Hebert CJ, Nonnemaker JM, Kim AE. Multiple tobacco product use among adults in the United States: cigarettes, cigars, electronic cigarettes, hookah, smokeless tobacco, and snus. Prev Med. 2014;62:14–19. 24440684 10.1016/j.ypmed.2014.01.014

[33] Kasza KA , Ambrose BK, Conway KP, et al. Tobacco-product use by adults and youths in the United States in 2013 and 2014. N Engl J Med. 2017;376(4):342–353. 28121512 10.1056/NEJMsa1607538 PMC5317035

[34] Chang JT , Levy DT, Meza R. Trends and factors related to smokeless tobacco use in the United States. Nicotine Tob Res. 2016;18(8):1740–1748. 26995793 10.1093/ntr/ntw090 PMC4941602

[35] Delnevo CD , Miller Lo E, Giovenco DP, et al.Cigar sales in convenience stores in the US, 2009-2020. JAMA. 2021;326(23):2429–2432. 34932088 10.1001/jama.2021.19692 PMC8693219

[36] Delnevo CD , Hrywna M, Foulds J, Steinberg MB. Cigar use before and after a cigarette excise tax increase in New Jersey. Addict Behav. 2004;29(9):1799–1807. 15530722 10.1016/j.addbeh.2004.04.024

[37] Gammon DG , Loomis BR, Dench DL, King BA, Fulmer EB, Rodgers T. Effect of price changes in little cigars and cigarettes on little cigar sales: USA, Q4 2011-Q4 2013. Tob Control. 2016;25(5):538–544. 26357952 10.1136/tobaccocontrol-2015-052343 PMC4903936

[38] National Cancer Institute and Centers for Disease Control and Prevention. Report on Smokeless Tobacco and Public Health: A Global Perspective. Washington, DC: National Cancer Institute and Centers for Disease Control and Prevention; 2014.

[39] Carpenter CM , Connolly GN, Ayo-Yusuf OA, Wayne GF. Developing smokeless tobacco products for smokers: an examination of tobacco industry documents. Tob Control. 2009;18(1):54–59. 18948390 10.1136/tc.2008.026583

[40] Mejia AB , Ling PM. Tobacco industry consumer research on smokeless tobacco users and product development. Am J Public Health. 2010;100(1):78–87. 19910355 10.2105/AJPH.2008.152603 PMC2791252

[41] Well Fargo Securities Equities Research. Nielsen: Tobacco All Channel Data Thru 5/18 - Cig Vol Declines Strengthen. New York: Wells Fargo and Company; 2019.

[42] Cantrell J , Huang J, Greenberg MS, Xaio H, Hair EC, Vallone D. Impact of e-cigarette and cigarette prices on youth and young adult e-cigarette and cigarette behaviour: evidence from a national longitudinal cohort. Tob Control. 2020;29(4):374–380. 31167900 10.1136/tobaccocontrol-2018-054764

[43] Pesko M , Courtemanche C, MacLean J. The effects of traditional cigarette and e-cigarette tax rates on adult tobacco product use. J Risk & Uncertainty. 2020;60(3):229–258. 10.1007/s11166-020-09330-9 PMC7880200 33584006

[44] U.S. Department of Health and Human Services. E-Cigarette Use Among Youth and Young Adults. A Report of the Surgeon General. Atlanta, GA: U.S. Department of Health and Human Services, Centers for Disease Control and Prevention, National Center for Chronic Disease Prevention and Health Promotion, Office on Smoking and Health; 2016.

[45] Huang J , Duan Z, Kwok J, et al. Vaping versus JUULing: how the extraordinary growth and marketing of JUUL transformed the US retail e-cigarette market. Tob Control. 2019;28(2):146–151. 29853561 10.1136/tobaccocontrol-2018-054382 PMC6274629

[46] Guillory J , Kim A, Murphy J, Bradfield B, Nonnemaker J, Hsieh Y. Comparing twitter and online panels for survey recruitment of e-cigarette users and smokers. J Med Internet Res. 2016;18(11):e288e288. 27847353 10.2196/jmir.6326 PMC5128722

[47] Levy DT , Tam J, Kuo C, Fong GT, Chaloupka F. The impact of implementing tobacco control policies: the 2017 tobacco control policy scorecard. J Public Health Manag Pract. 2018;24(5):448–457. 29346189 10.1097/PHH.0000000000000780 PMC6050159

[48] Huang J , Gwarnicki C, Xu X, et al. A comprehensive examination of own- and cross-price elasticities of tobacco and nicotine replacement products in the U.S. Prev Med. 2018;117:107–114. 29684418 10.1016/j.ypmed.2018.04.024 PMC6195827

[49] Zheng Y , Zhen C, Dench D, Nonnemaker JU. Demand for tobacco products in a system framework. Health Econ. 2016;17(8):1067–1086. 10.1002/hec.3384 27402419

[50] Tuchman A. Advertising and demand for addictive goods: the effects of e-cigarette advertising. Marketing Sci. 2019;38(6):994–11022.

[51] Cowen Research. Cowen’s Cigarette Global Guidebook. NY: Cowen Equity Research; 2018.

[52] Wells Fargo Securities. Nielsen: Tobacco “All Channel” Data Through 2/27. NY: Wells Fargo Securities Equity Research; 2017.

[53] Truth Initiative. Spinning-new-tobacco-industry-how-big-­tobacco-trying. https://truthinitiative.org/research-resources/tobacco-industry-marketing/spinning-new-tobacco-industry-how-big-tobacco-trying. Published 2021. Accessed December 29, 2021.

[54] NewYork Times. Vaping Is Big Tobacco’s Bait and Switch. https://www.nytimes.com/2019/03/08/opinion/editorials/vaping-ecigarettes-nicotine-safe.html. Published 2019. Accessed December 28, 2021.

[55] Center for Tobacco Free Kids. Take Down Tobacco. CTFK Web site. https://www.takingdowntobacco.org/ Published 2021. Accessed December 28, 2021.

[56] Glantz SA , Bareham DW. E-cigarettes: use, effects on smoking, risks, and policy implications. Annu Rev Public Health. 2018;39(1):215–235. 29323609 10.1146/annurev-publhealth-040617-013757 PMC6251310

[57] Herzog B. Nielsen: Tobacco “All Channel” Data - 9/9. New York: Wells Fargo Securities; 2017.

[58] Wells Fargo Securities. Nielsen: Tobacco “All Channel” Data 3/24. NY: Well Fargo Equity Research; April 3, 2018.

[59] Wells Fargo Securities. Nielsen: Tobacco All Channel Data Thru 9/7 - Cig Vol Declines Hold Steady Recent Neg E-Cig News May Be Having Impact. NY: Well Fargo Equity Research; 2019.

[60] Altria website. Altria Makes $12.8 Billion Minority Investment in JUUL to Accelerate Harm Reduction and Drive Growth. http://investor.altria.com/press-releases/news-details/2018/altria-makes-12.8-billion-minority-investment-injuul-toaccelerate-harm-reduction-and-drive-growth/default.aspx. Accessed February 20, 2023.

[61] University of Bath. Tobacco Tactics: E-cigarettes. https://tobaccotactics.org/wiki/e-cigarettes/. 2021. Accessed December 30, 2021.

[62] Ali FRM , Diaz MC, Vallone D, et al. E-cigarette unit sales, by product and flavor type - United States, 2014-2020. MMWR Morb Mortal Wkly Rep. 2020;69(37):1313–1318. 32941416 10.15585/mmwr.mm6937e2 PMC7498168

[63] CNBC. Altria launches Iqos tobacco device in US, and the timing couldn’t be better. CNBC News. https://www.cnbc.com/2019/10/04/altria-launches-iqos-tobacco-device-in-us-and-the-timing-couldnt-be-better.html. 2019. Accessed January 5, 2020.

[64] Barklays Equity Research. Altria Group Inc./ British American Tobacco Plc: The lose-lose patent disputes. UK: Barklay; October 1, 2021.

[65] Bloomberg. Altria Enters Growing Oral Nicotine Products Category with on! Pouch Product. 2019. at https://www.bloomberg.com/press-releases/2019-06-03/altria-enters-growing-oral-nicotine-products-category-with-on-pouch-product. Accessed December 11, 2021.

[66] Centers for Disease Control and Prevention. National Center for Health Statistiics, National Health Interview Survey. 2022. https://ftp.cdc.gov/pub/Health_Statistics/NCHS/NHIS/SHS/2017_SHS_Table_A-12.pdf. Accessed February 22, 2023.

[67] Levy DT , Sánchez-Romero LM, Travis N, et al. US nicotine vaping product simsmoke simulation model: the effect of vaping and tobacco control policies on smoking prevalence and smoking-attributable deaths. Int J Environ Res Pub Health. 2021;18(9):4876. 34063672 10.3390/ijerph18094876 PMC8124578

[68] Levy DT , Sanchez-Romero LM, Li Y, et al. England SimSmoke: the impact of nicotine vaping on smoking prevalence and smoking-attributable deaths in England. Addiction. 2021;116(5):1196–1211. 32949419 10.1111/add.15269 PMC9364758

[69] Meza R , Jimenez-Mendoza E, Levy DT. Trends in tobacco use among adolescents by grade, sex, and race, 1991-2019. JAMA Netw Open. 2020;3(12):e2027465e2027465. 33263760 10.1001/jamanetworkopen.2020.27465 PMC7711321

[70] Levy DT , Warner KE, Cummings KM, et al. Examining the relationship of vaping to smoking initiation among US youth and young adults: a reality check. Tob Control. 2019;28(6):629–635. 30459182 10.1136/tobaccocontrol-2018-054446 PMC6860409

[71] Fama E , French K. Profitability, investment and average return. J Fin Econ. 2006;82(3):491–518.

[72] Fama E. Efficient capital markets: a review of theory and empirical work. J Finance. 1970;25(2):383–417.

[73] Sharpe W. Investments. New Jersey: Prentice-Hall; 1978.

[74] Fama E , Fisher L, Jensen M, Roll R. The adjustment of stock prices to new information. Int Econ Rev. 1969;10(2):1–21.

[75] Andersen M , Bauhoff S. The share price effect of CVS health’s announcement to stop selling tobacco: a comparative case study using synthetic controls. Forum Health Econ Policy. 2016;20(1). 10.1515/fhep-2015-0045 31419901

[76] Sloan FA , Mathews CA, Trogdon JG. Impacts of the master settlement agreement on the tobacco industry. Tob Control. 2004;13(4):356–361. 15564618 10.1136/tc.2003.007229 PMC1747950

[77] Sloan FA , Trogdon JG, Mathews CA. Litigation and the value of tobacco companies. J Health Econ. 2005;24(3):427–447. 15811537 10.1016/j.jhealeco.2004.09.009

[78] Starr MA , Drake K. Graphic warning labels and the demand for cigarettes. Tob Control. 2017;26(2):169–174. 27015879 10.1136/tobaccocontrol-2015-052775

[79] Branston JR , Gilmore AB. The failure of the UK to tax adequately tobacco company profits. J Public Health (Oxf). 2020;42(1):69–76. 30726968 10.1093/pubmed/fdz004 PMC7044670

[80] Ciura B. Sure Dividend: High-Quality Dividend Stocks, Long-Term Plan. https://www.suredividend.com/best-tobacco-stock/. 2022. Accessed January 7, 2022.

[81] Backinger CL , Meissner HI, Ashley DL. The FDA “deeming rule” and tobacco regulatory research. Tob Regul Sci. 2016;2(3):290–293. 29423429 10.18001/TRS.2.3.8 PMC5800318

[82] Gottlieb S , Zeller M. A nicotine-focused framework for public health. N Engl J Med. 2017;377(12):1111–1114. 28813211 10.1056/NEJMp1707409

[83] Credit Suisse GER. Global Tobacco High-single-digit EPS growth at risk. London: Credit Suisse Securities; 2018.

[84] Food and Drug Administration. Deeming tobacco products to be subject to the federal food, drug, and cosmetic act, as amended by the family smoking prevention and tobacco control act; regulations on the sale and distribution of tobacco products and required warning statements for tobacco products. Fed Regist. 2016;81:28973–29106. 27192730

[85] Food and Drug Administration. The Federal Response to the Epidemic of E-Cigarette Use, Especially Among Children, And the Food and Drug Administration’s Compliance Policy. https://www.fda.gov/news-events/congressional-testimony/federal-response-epidemic-e-cigarette-use-especially-among-children-and-food-and-drug. 2019. Accessed January 7, 2022.

[86] Food and Drug Administration. Pre-Market Tobacco Product Marketing Granted Orders. https://www.fda.gov/tobacco-products/premarket-tobacco-product-applications/premarket-tobacco-product-marketing-granted-orders. 2021. Accessed December 12, 2021.

[87] Food and Drug Administration. FDA Authorizes Marketing IQOS Tobacco Heating System Reduced Exposure Information. https://www.fda.gov/news-events/press-announcements/fda-authorizes-marketing-iqos-tobacco-heating-system-reduced-exposure-information. 2021. Accessed December 12, 2021.

[88] Ratajczak A , Jankowski P, Strus P, Feleszko W. Heat not burn tobacco product-a new global trend: impact of heat-not-burn tobacco products on public health, a systematic review. Int J Environ Res Public Health. 2020;17(2):409. 31936252 10.3390/ijerph17020409 PMC7014072

[89] Stoklosa M , Cahn Z, Liber A, Nargis N, Drope J. Effect of IQOS introduction on cigarette sales: evidence of decline and replacement. Tob Control. 2020;29(4):381–387. 31209129 10.1136/tobaccocontrol-2019-054998

[90] University of Bath. Addiction at Any Cost: Philip Morris International Uncovered. Bath: University of BATH; 2020.

[91] British American Tobacco. Modern oral products. bat.com/snus. 2021. Accessed February 22, 2023.

[92] Barclays Equity Research. Swedish Match: Q321 to suffer from tough comps. UK: Barklays; 2021.

[93] Jennifer M. Swedish match agrees to $16 billion takeover by Philip Morris [press release]. NY: Wall Street Journal; 2022.

[94] Burki TK. Philip Morris international purchases vectura. Lancet Respir Med. 2021;9(12):e122. 34600613 10.1016/S2213-2600(21)00445-8

[95] Cahn Z , Drope J, Douglas CE, et al. Applying the population health standard to the regulation of electronic nicotine delivery systems. Nicotine Tob Res. 2021;23(5):780–789. 32960217 10.1093/ntr/ntaa190 PMC8095236

[96] US Food and Drug Admin. Premarket Tobacco Product Applications. FDA. https://www.fda.gov/tobacco-products/market-and-distribute-tobacco-product/premarket-tobacco-product-applications. 2021. Accessed December 11, 2021.

[97] Wells Fargo Securities. Good For the Goose, Less For the Gander. NY: Wells Fargo Equity Research; 2016.

[98] Wells Fargo Securities. Final Deeming E-Cigs Regs Released--Quick Take. NY; Well Fargo Equity Research; 2016.

[99] US Food and Drug Admin. FDA Permits Marketing of E-Cigarette Products, Marking First Authorization of Its Kind by the Agency. FDA. https://www.fda.gov/news-events/press-announcements/fda-permits-marketing-e-cigarette-products-marking-first-authorization-its-kind-agency. 2021. Accessed December 10, 2021.

[100] DeCicca P , Kenkel D, Lovenheim MF. The economics of tobacco regulation: a comprehensive review. J Econ Lit. 2022;60(3):883–970. 37075070 10.1257/jel.20201482 PMC10072869

[101] Chaloupka FJ , Straif K, Leon ME; Working Group, International Agency for Research on Cancer. Effectiveness of tax and price policies in tobacco control. Tob Control. 2011;20(3):235–238. 21115556 10.1136/tc.2010.039982

[102] Levy DT , Meza R, Yuan Z, et al. Public health impact of a US ban on menthol in cigarettes and cigars: a simulation study. Tob Control. Published Online First: 02 September 2021. 10.1136/tobaccocontrol-2021-056604 PMC9210349 34475258

[103] US Federal Trade Commission. Cigarette Report For 2020. FTC. https://www.ftc.gov/system/files/documents/reports/federal-trade-commission-cigarette-report-2020-smokeless-tobacco-report-2020/p114508fy20cigarettereport.pdf. 2021. Accessed December 12, 2021.

[104] Levy D , Liber A, et al. Follow the money: a closer look at us tobacco industry marketing expenditures. Tob Control. 2022. 10.1136/tobaccocontrol-2021-056971 PMC9346571 35074930

[105] Politico. Tobacco lawsuits could upend Biden’s plan for historic menthol ban. https://www.politico.com/news/2021/11/14/tobacco-lawsuits-biden-menthol-ban-521174. 2021. Accessed December 14, 2021.

[106] Gilmore AB , Fooks G, Drope J, Bialous SA, Jackson RR. Exposing and addressing tobacco industry conduct in low-income and middle-income countries. Lancet. 2015;385(9972):1029–1043. 25784350 10.1016/S0140-6736(15)60312-9 PMC4382920

[107] Mathers A , Hawkins B, Lee K. Transnational tobacco companies and new nicotine delivery systems. Am J Public Health. 2019;109(2):227–235. 30571303 10.2105/AJPH.2018.304813 PMC6336036

[108] Chaloupka FJ , Sweanor D, Warner KE. Differential taxes for differential risks--toward reduced harm from nicotine-yielding products. N Engl J Med. 2015;373(7):594–597. 26267620 10.1056/NEJMp1505710

[109] Gaca M , Williamson J, Digard H, Adamson L, Hawkridge L, Proctor C. Accelerating regulatory acceptance of reduced-risk tobacco and nicotine products. Nicotine Tob Res. 2022;24(9):1371–1378. 35171296 10.1093/ntr/ntac041 PMC9356683

