# Supplementary material for: Do Tobacco Companies Have an Incentive to Promote “Harm Reduction” Products?: The Role of Competition
Source: Nicotine Tob Res. 2023 Jan 24;25(12):1810–21. doi: 10.1093/ntr/ntad014 (PMC10664083; doi:10.1093/ntr/ntad014)
Supplement: ntad014_suppl_Supplementary_Figures [file ntad014_suppl_supplementary_figures.pdf]

Supplement One: Figures

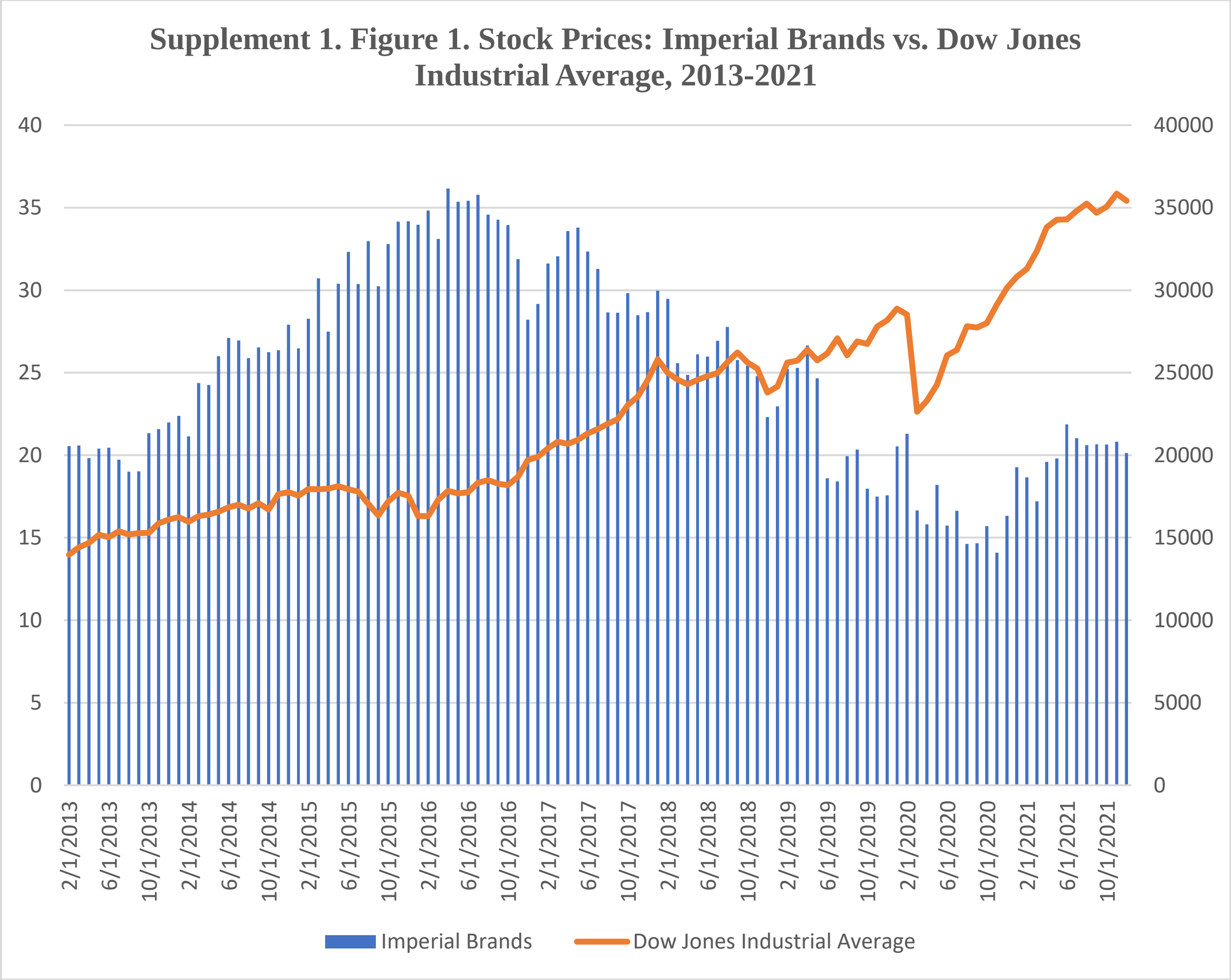

Supplement 1. Figure 2. Stock Prices: Philip Morris International vs. Dow Jones Industrial Average, 2013-2021

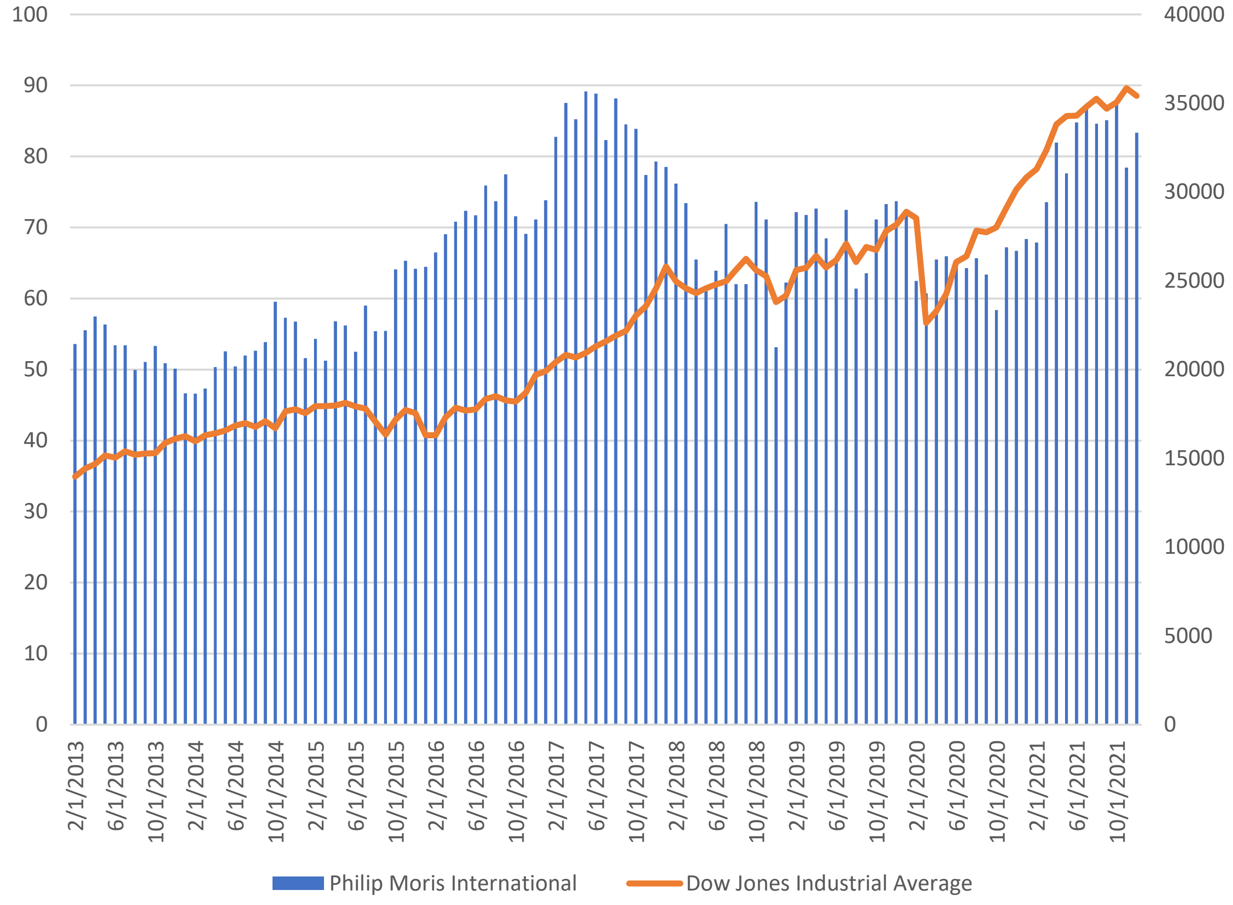

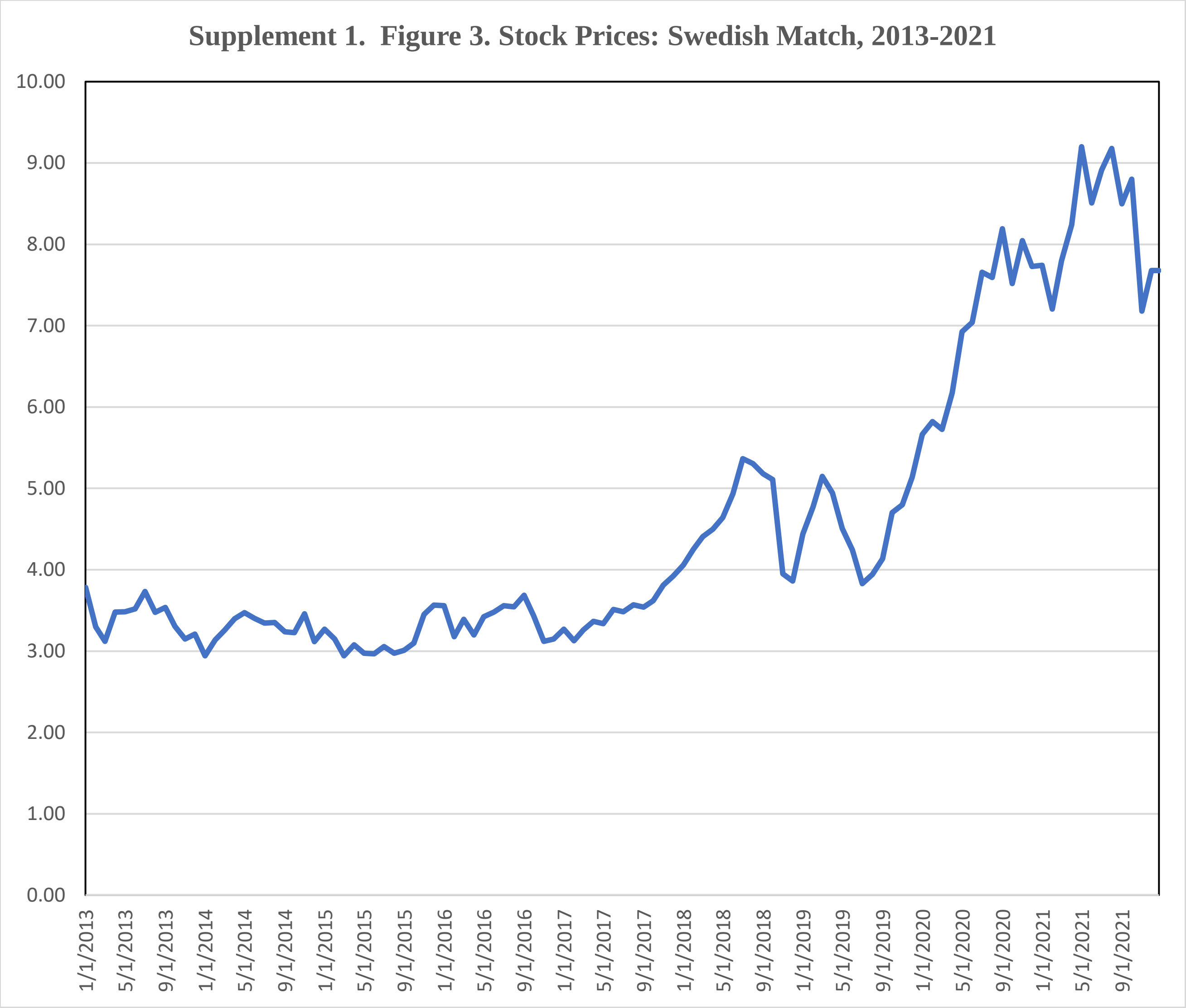
Supplement 1. Figure 3. Stock Prices: Swedish Match, 2013-2021
10.00
9.00
8.00
7.00
6.00
5.00
4.00
3.00
2.00
1.00
0.00
1/1/2013
5/1/2013
9/1/2013
1/1/2014
5/1/2014
9/1/2014
1/1/2015
5/1/2015
9/1/2015
1/1/2016
5/1/2016
9/1/2016
1/1/2017
5/1/2017
9/1/2017
1/1/2018
5/1/2018
9/1/2018
1/1/2019
5/1/2019
9/1/2019
1/1/2020
5/1/2020
9/1/2020
1/1/2021
5/1/2021
9/1/2021
